# Paediatric Systemic Lupus Erythematosus with chronic diarrhea as an initial presentation

**DOI:** 10.12669/pjms.41.4.10235

**Published:** 2025-04

**Authors:** Ali Abdur Rahman, Asfand Tariq

**Affiliations:** 1 Ali Abdur Rahman, Department of Paediatric Medicine, Shaikh Zayed Hospital, Lahore, Pakistan; 2 Asfand Tariq, Department of Paediatric Medicine, Shaikh Zayed Hospital, Lahore, Pakistan

**Keywords:** Childhood Systemic lupus erythematosus, Lupus enteritis, Chronic diarrhea

## Abstract

Lupus enteritis (LE) is a rare manifestation of Childhood Systemic lupus erythematosus (cSLE). The presentation is non-specific i.e loose motions, non-specific abdominal pain, nausea and vomiting. Presenting as a sole initial clinical finding in the absence of other common findings of systemic lupus erythematosus (SLE) makes the diagnosis challenging. After a detailed workup, our patient was diagnosed with LE based on history, physical and laboratory investigations and responded well with initial management. Our report emphasizes the rarity of LE and the significance of its prevalence in the childhood population due to increased morbidity, mortality and complications such as intestinal bleeding, perforation and infarction. With early diagnosis and treatment, there is an excellent prognosis.

## INTRODUCTION

Systemic lupus erythematosus (SLE) is a multisystem, autoimmune inflammatory disease. SLE affects 161000 to 322000 people in the United States, involving any organ, including the gastrointestinal tract in approximately 20% of children having abdominal pain as a frequent symptom.^1^ Lupus enteritis (LE) is a rare manifestation of SLE that affects 0.2% to 5.8% of these patients making the diagnosis difficult, especially in the absence of other SLE symptoms.^1^ The incidence of Childhood SLE (cSLE) range between 0.36 and 2.5 per 100,000 children, a prevalence of 1.89-34.1 per 100,000, with median age of onset for childhood SLE (cSLE) 11-12 years and rare involvement of children less than five years.^2^,

According to the classification criteria by the American College of Rheumatology (ACR), four out of 11 should be satisfied, and by Systemic Lupus International Collaborating Clinics (SLICC) the fulfilment of at least four out of 17 criteria is required, with at least one clinical and one laboratory criterion or renal histology that is compatible with lupus nephritis plus ANA or anti-DNA positive antibodies.^3^ Gastrointestinal (GI) involvement is rare in cSLE and scarce data have been reported as there are only a few case reports mentioning lupus enteritis as the only initial presentation of active SLE.^3^

Other common findings of cSLE are fever, malar or butterfly rash, cutaneous vasculitis, leucopenia, thrombocytopenia and also involves liver, kidneys and central nervous system.^3^,^4^ LE or lupus mesenteric vasculitis (LMV) is an immune complex-mediated inflammatory vasculitis, due to deposition and thrombosis of the intestinal vessels, related to antiphospholipid antibodies.^5^ LE varies from mild, non-specific abdominal pain, nausea, vomiting and diarrhoea to severe gastrointestinal bleeding.^5^ However, it is usually reported in patients with well-established SLE.^5^ Additionally, it is exceedingly rare for lupus enteritis to be the sole initial presentation of paediatric-onset SLE. 5

## CASE PRESENTATION

A 12 years old South-Asian girl was brought to the outpatient department at Shaikh Zayed Hospital, Lahore with recurrent, sharp, non-radiating, right lower quadrant abdominal pain, watery stools, without mucus or blood, up to five times per day, for one week with similar history, off and on for past two years. It was not associated with any aggravating factors; however, it was relieved after passing loose stools. She also had generalized swelling of the body particularly in the periorbital region, hands up to forearms and feet up to knees for the past two months. She reported a low-grade fever of up to 100 °F off and on for the past six months without night sweats, an undocumented weight loss over the past year with difficulty in gaining weight and lean body mass.

**Table-I:** Baseline investigations.

Investigation	Patient value	Reference value
Hemoglobin	6.9	11.5-14.5 g/dl
Total Red blood cells	2.5	4.0-5.3 x 10^12^/L
Hematocrit	21	33-43 %
Mean corpuscular volume (MCV)	84	76-90 fL
White blood cells	2.7	4.0-12 x 10^9^/L
Neutrophils	60.9%	32-62%
Lymphocytes	30.7%	46-55%
Platelets	167	150-400 x 10^9^/L
Reticulocyte count	1.4%	0.20-2.0%
Erythrocyte sedimentation rate (ESR)	100	<15 mm/1^st^ hour
Random Glucose	72	70-200 mg/dl
Creatinine	0.54	0.55-1.02 mg/dl
Blood urea nitrogen	15	8-22 mg/dl
Uric acid	4.2	2.6-6.0 mg/dl
Alanine aminotransferase	21	<34 U/L
Aspartate aminotransferase	35	<31 U/L
Alkaline phosphatase	151	64-359 U/L
Total bilirubin	0.2	0.1-1.2 mg/dl
Gamma-glutamyl transferase	15	< 33
Total serum protein	5.4	6.0-8.5
Serum Albumin	1.6	3.5-5.2
Serum Globulins	3.8	1.8-3.5
Additional investigations:		
Anti-tissue transglutaminase IgA	< 0.5	<10 AU/ml
Urine protein (spot)	1.3	< 14.0 mg/dl
Urine creatinine (spot)	35 mg/dl	-
Protein/Creatinine Ratio	0.04	< 0.2mg/mg
Blood in urine	++	-
Red blood cells in urine	18-20/HPF	-
Pus cells	8-10/HPF	-
Serum Complement-3 (C3)	17	82-173 mg/dl
Anti-nuclear antibodies (ANA)	Positive	-
Anti-Double strand DNA (anti-ds-DNA) antibodies	Positive	-
Renal Biopsy	Mesangioproliferative glomerulonephritis	

There was no history of chronic cough, hair loss, joint pains, morning stiffness, rash (including malar rash), oral ulcers, respiratory difficulty or chest pain, and no blood, froth, pus or pain with urine. She did not report any recent change in diet, history of travel, or tuberculosis contact. There was no family history of any rheumatological disease, thyroid illness, hepatitis, or dietary/gluten allergy. On examination, the girl was well-oriented in time, place and person. Her weight was 28 kg (below the third centile), height was 140 cm (tenth centile). Her sexual maturity ratings (SMRs) were tanner stage-III. She was vitally stable. Systemic examination revealed pallor with prominent periorbital swelling, pitting edema on hands and feet up to knees without any visible bruises or petechiae on skin. On chest auscultation, there was decreased air entry at the right lung base. The abdomen was soft, with mild tenderness in the right lower quadrant, audible bowel sounds, no rebound tenderness, no shifting dullness, no fluid thrill or palpable visceromegaly. Sensory and neurological examination was normal. The investigations are shown in the table below:

Based on the history, clinical examination and baseline investigations, a differential diagnosis of Celiac disease, protein-losing enteropathy, inflammatory bowel disease, Systemic Lupus Erythematosus, and Intestinal tuberculosis was made. Based on the additional investigations including the renal biopsy, the patient was diagnosed with SLE, and treatment was initiated with pulse intravenous methylprednisone for three days followed by continued use of tablets of deltacortil (2mg/kg/day), Mycophenolate mofetil, hydroxychloroquine, lisinopril and norvasc.

## DISCUSSION

Our case reports the uncommon presentation of cSLE in that despite being a rare manifestation, LE can be an initial presentation in cSLE. Due to its heterogeneity, it is not included in the diagnostic criteria of SLE. Therefore, it is of significance in children with chronic gastrointestinal manifestations due to associated mortality and morbidity risk from bowel infarction and perforation.

Typically cSLE presents with fever, malar rash, hematological, renal and CNS manifestations which also included in the diagnostic criteria of SLE. As mentioned in a study by Chowichian M et al. 16.7-27.5% of paediatric-onset SLE patients have initial gastrointestinal manifestations, most commonly abdominal pain which is rarely seen as the sole initial manifestation.^5^ It also mentions a single case of LE in 69 children with SLE marking its rarity in children.^5^

Our case is most unusual since the child presented with recurrent abdominal pain associated with diarrhea over the past one year without other clinical manifestations. Only after the baseline and additional investigations, the diagnostic criteria for SLE was completed. Initial management with intravenous methylprednisolone, immunosuppressive agents (mycophenolate mofetil), hydration, bowel rest and supportive therapy resulted in clinical improvement after one week.

Compared to adult-onset SLE, cSLE is more aggressive, with higher disease activity and medication burden (including corticosteroids and other immunosuppressive drugs) that contributes to the increased morbidity and mortality, the presence of greater damage at the time of diagnosis, and a higher incidence of renal, cardiovascular and neuropsychiatric involvement.^6^ GI manifestations of lupus are rare and LE may be prevalent in about 0.2-9.7% of patients with SLE; however, LE presenting as the initial manifestation is quite rare, and diagnosing without a prior background of SLE can be challenging.^7^-^8^

Similar facts are reinstated by another study by Raees A et al. with additional emphasis on the most commonly involved sites - the jejunum and ileum followed by the colon, duodenum, and rectum, respectively.^8^ Demonstration of bowel wall thickness greater than three millimeter (Target sign), mesenteric vessel engorgement (Combs sign), and attenuation of mesenteric fat are the classical features of LE on imaging.^8^ There is an excellent prognosis since there is an adequate response to steroid and the immunosuppresive therapy; However, there is a risk of relapse after an initial improvement in up to 23% of cases.^8^ Due to the high fatality rate from LE-mediated bowel infarction, perforation and bleeding, the suspicion of such disease should be kept high in children with chronic gastrointestinal manifestations. 9

## CONCLUSION

LE may present as the sole initial manifestation of cSLE. Due to the non-specific and heterogenous nature of gastrointestinal manifestations, such clinical findings can be overlooked while suspecting SLE in the absence of other common findings: fever, malar rash, arthritis, hematological and renal findings. Due to the high morbidity and mortality risk from intestinal bleeding, perforation and infarction, close observation should be instituted in such patients, as early recognition and management leads to excellent prognosis.
